# Wetting of a Stepped Platinum (211) Surface

**DOI:** 10.1021/acs.jpcc.2c08360

**Published:** 2023-03-01

**Authors:** K. Mistry, N. Gerrard, A. Hodgson

**Affiliations:** Surface Science Research Centre and Department of Chemistry, University of Liverpool, Liverpool L69 3BX, U.K.

## Abstract

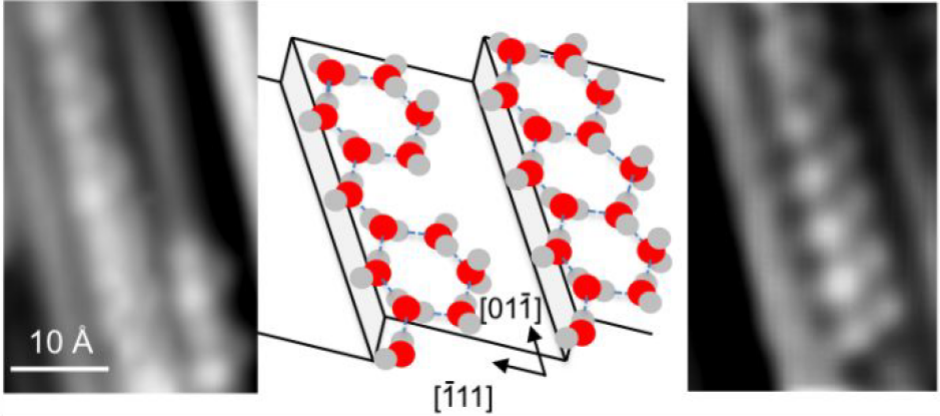

Steps stabilize water adsorption on metal surfaces, providing favorable binding sites for water during wetting or ice nucleation, but there is limited understanding of the local water arrangements formed on such surfaces. Here we describe the structural evolution of water on the stepped Pt(211) surface using thermal desorption, low-energy electron diffraction, and scanning tunneling microscopy to probe the water structure. At low coverage water forms linear structures comprising zigzag chains along the steps that are decorated by H-bonded rings every one or two units along the terrace. Simple 2-coordinate H-bonded chains are not observed, indicating the Pt step binds too weakly to compensate entirely for a low water H-bond coordination number. As the coverage increases, water chains assemble into a disordered (2 × 1) structure, likely made up of the same narrow water chains along the steps with little or no H-bonding between adjacent structures. The chain structure disappears as water adsorption saturates the surface to form an incommensurate, disordered network of water rings of different size. Although the steps on Pt(211) clearly stabilize water adsorption and direct growth, the surface does not support the simple 1D chains previously proposed or an ordered 2D network such as seen on other surfaces. We discuss reasons for this and the factors that determine the behavior of the first water layer on stepped metal surfaces.

## Introduction

Platinum is an effective redox catalyst for the formation of water in electrochemical fuel cells, stimulating considerable interest in how it interacts with water, OH, and other intermediate species.^1,2^ As a result, a number of studies have investigated how water binds on the close-packed Pt surface, revealing details of how the film nucleates,^3,4^ the complex mix of pentamer, hexamer, and heptamer rings that stabilizes the first layer of water,^4−8^ and how the structure of this layer influences the growth and crystallization of multilayer films.^9−12^ The importance of surface steps as nucleation sites was identified in the earliest STM studies, with water forming narrow “quasi-one-dimensional chains” less than 10 Å wide on the top edge of Pt steps, with more extended 2D islands nucleating on the lower terrace.^3^ Steps were also found to stabilize water on other metal surfaces,^13−16^ and this, along with the importance of low coordinate sites for practical catalysts,^17−19^ has spurred experiments to examine how step sites influence water adsorption on Pt.^20−26^ The general conclusion is that low coordinate metal steps enhance the binding energy of water and stabilize adsorption, but there is less clarity about the precise structures formed on different surfaces or indeed the amount of water present.

One common interpretation of the stabilizing effect of steps is that water may be bound as a linear 1D chain along the step edges, and support for this idea comes from several sources. For example, Grecea et al.^20^ found that the binding energy of water on Pt(533) decreased once the coverage was sufficient to saturate the step sites, with further water adsorbing to saturate the surface layer having a slightly lower binding energy.^20,22^ The increased binding energy at the step sites is reproduced by DFT calculations,^20,27,28^ suggesting that adsorption occurs via stabilization of 1D chains above the steps, followed by weaker adsorption on the narrow (111) terraces to complete the water layer.^23^ Vibrational sum-frequency generation experiments suggest water adopts an H-down arrangement in 1D water chains at low coverage and that this orientation persists as water forms a more extended network at higher coverage.^29^ On Pt(211), where the (111) terraces are slightly narrower, surface X-ray diffraction and near-edge X-ray adsorption fine structure (NEXAFS) measurements find two distinct O sites above the step Pt atoms,^30,31^ supporting the idea that water is stabilized by formation of 1D zigzag water chains, as suggested by calculations.^27,28,32−36^

Despite the general conclusion that steps stabilize water, direct experimental evidence of the local water binding geometry adopted on Pt steps is limited. Moreover, calculations suggest that simple 1D chains at the step edge are not thermodynamically stable because addition of further water to these chains to form ring structures increases the average water binding energy on Pt(533).^28^ A recent STM study reported double-stranded water rings were formed on Pt(553) in preference to 2-coordinate chains,^26^ even though these (111) steps are expected to bind water more tightly than (100) steps.^35^ Linear 2-coordinate water chains have been observed on other surfaces by STM, with water forming flat zigzag chains that saturate Ni(110) at a coverage of just 0.5 water/Ni.^37^ Chain formation is driven by the strong Ni–water bond, which mitigates the low water H-bond coordination number in the chains, and by the short Ni surface lattice repeat, which hinders formation of ring structures. Despite the stabilization provided by adsorption at step sites on Pt, it is not clear if the binding energy at steps is sufficient to overcome the reduced H-bond coordination and stabilize simple 1D water chains on Pt(211);^30,31^ it may instead be preferable to incorporate these adsorption sites into a 2D water network, gaining the benefit of both a higher H-bond coordination and the stable Pt step sites. On Cu(511) wetting is driven by the presence of both strongly binding step sites and more weakly adsorbing terrace sites, water forming 2D commensurate structures, including a hexagonal structure with the mixture of buckled H-up and H-down orientations required to sustain ice growth.^38,39^ In the case of Pt(211), which has a higher binding energy for water and larger step spacing than Cu(511), it is not yet clear how steps influence water nucleation or the nature of any extended 2D wetting layer.

In this study we re-examine water adsorption on Pt(211) using temperature-programmed desorption (TPD), low-energy electron diffraction (LEED), and scanning tunneling microscopy (STM) to examine the structures formed. We do not find the simple 1D chains commonly anticipated; instead, we find that water forms chains with a double period along the step that are H-bonded into water rings on the adjacent terrace. These structures show some disorder along the terrace but frequently form regular chains of rings, probably in the form of isolated or linked hexagons. Further water adsorption saturates the steps with water, forming a disordered (2 × 1) structure, with strong ordering along the steps but disorder between adjacent steps, indicating any H-bond linkage between adjacent chains is weak. Increasing the water coverage to saturate the first layer replaces the chains along the steps with a disordered 2D H-bond network containing rings of different size. Although the propensity for water to adsorb atop the steps as zigzag chains is clear, the Pt–water step interaction is not sufficiently strong that this dominates adsorption sufficiently to force water into simple 2-coordinate chains.

## Experimental Methods

The Pt(211) sample was prepared in an ultrahigh-vacuum environment (*P* < 1 × 10^–10^ mbar) by repeated cycles of Ar^+^ ion sputtering followed by annealing to 1000 K to reorder the Pt surface. Oxygen treatment was also used during the initial cleaning period to remove any carbon build up on the surface. The resulting surface showed a sharp LEED pattern, with STM imaging the steps as regular high contrast lines spaced 6.8 Å apart across the (211) terraces. Experiments were performed in two separate UHV systems. The STM comprised a preparation chamber and dewar-type SPM system (Createc), operated at 80 K during imaging.^40^ The step direction was determined from the location of added Pt rows at the edge of the (211) terraces. Water (99.9 atom % D_2_O) was degassed by repeated vacuum distillation and deposited directly onto the surface, held at 80 to 140 K, using an effusive (300 K) directional doser, then annealed to different temperatures to allow it to order prior to imaging. STM images were recorded in constant current mode at 80 K with an electrochemically etched tungsten tip. No significant differences in water arrangement were found for deposition or anneal temperatures between 120 and 160 K, where multilayer water can freely desorb, indicating the structures reported here are thermodynamically stable.

LEED and thermal desorption spectroscopy (TDS) were performed in a second system. The sample was mounted directly to a cryostat via two Ta wires that are used for heating, allowing the surface to be heated at controlled rates up 20 K s^–1^. Water was deposited directly into the front surface using a collimated effusive (300 K) molecular beam, and sticking or desorption was detected using a quadrupole mass spectrometer. Immediately prior to these experiments, the flux of the molecular beam was calibrated against the dose required to complete the hexagonal water structure formed on Cu(511),^38^ which has a water coverage of 1.18 × 10^15^ water cm^–2^, very close to that of an ice I_h_(0001) layer, and is here termed a dose of one monolayer (1 ML) water. Because the sticking probability for water on all metal surfaces is close to unity at 80 K,^41^ we expect this calibration to provide a good estimate of the water coverage on Pt(211). LEED measurement of surface ordering were made using a dual-MCP amplified LEED system (OCI), operated at <5 nA to minimize electron damage to water structures.^42^ We note that previous experiments in the same two STM and TPD/LEED chambers have demonstrated consistent results between the two chambers, finding new ordered water structures on Cu(511)^38,39^ and Ni(110)^37,43^ and reproducing the ordered structures found on SnPt(111) alloy surfaces,^40,44^ without any evidence for formation of metastable phases or differences between the two deposition chambers.

## Results and Discussion

**Figure 1:**
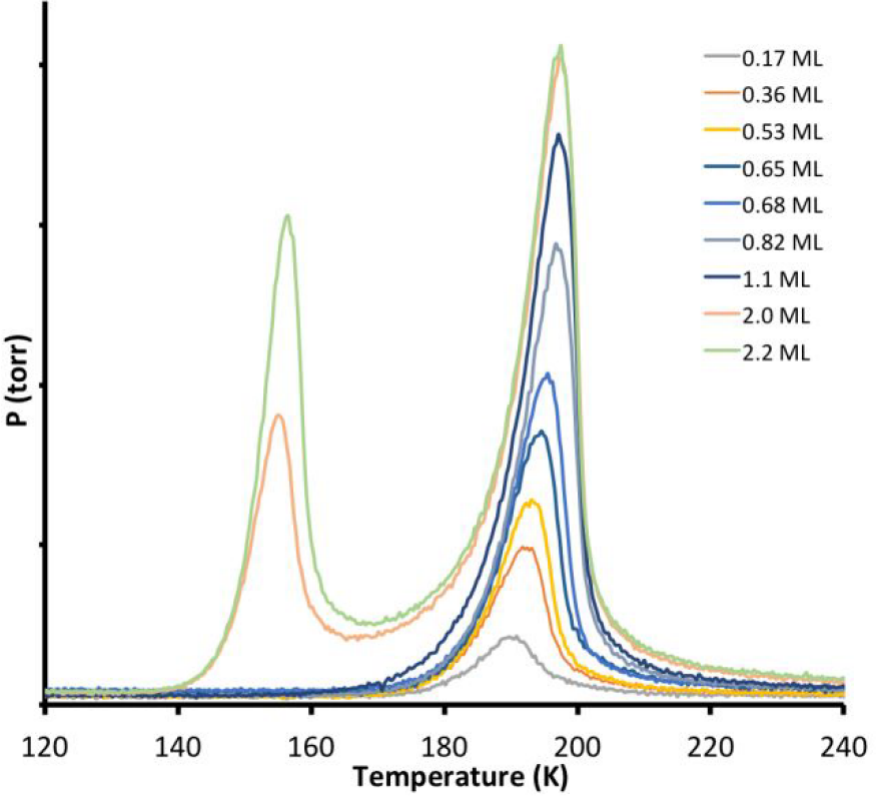
Thermal desorption of water (D_2_O) from the Pt(211) surface, recorded at a heating rate of 1 K s^–1^. The thermal desorption spectra show two peaks: a multilayer peak near 150 K and the monolayer peak just below 200 K. The water coverage indicated in the legend on the right is that obtained by calibration of the beam flux required to complete the 2D hexagonal water monolayer on Cu(511), defined as 1 ML water here.

Water adsorption on the Pt(211) surface resulted in the thermal desorption spectra shown in Figure 1. The spectra show two peaks: the first peak appearing near 190 K, associated with water stabilized by the Pt surface, followed by a second peak near 150 K which continues to grow indefinitely as the water dose is increased and is associated with multilayer water. As reported earlier,^25^ we find no evidence of a second surface peak intermediate between the surface peak and the multilayer peak, which contrasts with surfaces where the steps are more separated that show an additional surface peak.^23^ Although we do find evidence that water dissociates during adsorption/desorption before the surface is properly clean and well-ordered, reducing the size and shifting the desorption peak during repeated exposure,^24^ dissociation ceases once the surface is clean and well-ordered, so that repeated TDS measurements are reproducible and depend only on the water coverage on the surface, not the prior history of water deposition. This behavior is similar to that found on Cu(110)^45,46^ and suggests that water desorption outcompetes dissociation on clean, well-ordered Pt(211) below 200 K but can be mediated by defects or impurities when these are present.

The surface-stabilized peak shows a common leading edge as the coverage is increased from 0.2 up to 0.8 ML, similar to the pseudo-zero-order desorption found for many other H-bonded water layers,^47^ with an activation energy to desorption of 52.4 ± 1 kJ mol^–1^. This coverage range extends beyond that where low-dimensional structures, such as 1D chains, could be responsible for adsorption to a point where water must be accommodated at sites other than the step alone, with no apparent change in water binding energy. Only as the water coverage is increased toward 1.1 ML, the coverage at which the surface peak saturates on Pt(211), does the leading edge of the desorption curve shift slightly to lower temperature, indicating a reduced binding energy, with the multilayer peak appearing above 1.1 ML water. On the basis of the desorption behavior, we conclude that Pt(211) binds water in structures that stabilize up to 1.8 water per Pt step atom, before the layer restructures to accommodate ca. 2.4 water per step atom at saturation with a slightly reduced binding energy.

The lateral order of the water layer was examined using LEED and showed bright Pt integer order beams with additional fractional order diffraction beams that indicate the presence of a partially ordered water layer (see Figure S1). Annealing ca. 0.4 ML to 160 K reveals diffuse diffraction spots at the half-order positions in the [011] direction, indicating limited two times ordering along the close-packed Pt steps. Further increasing the coverage to ca. 0.7 ML caused the diffraction spots at the half-order positions to become increasingly faint and streaked in the [111] direction, perpendicular to the steps, suggesting the order in the wetting layer has reduced. The additional diffraction features disappear entirely at high coverage as water saturates the surface. The LEED data suggest that we have order along the step direction on the Pt(211) surface with a two-unit repeat at low or intermediate coverage, followed by increased disorder as the water layer completes—conclusions that are mirrored in the STM images found for water on the Pt(211) surface, described below.

**Figure 2:**
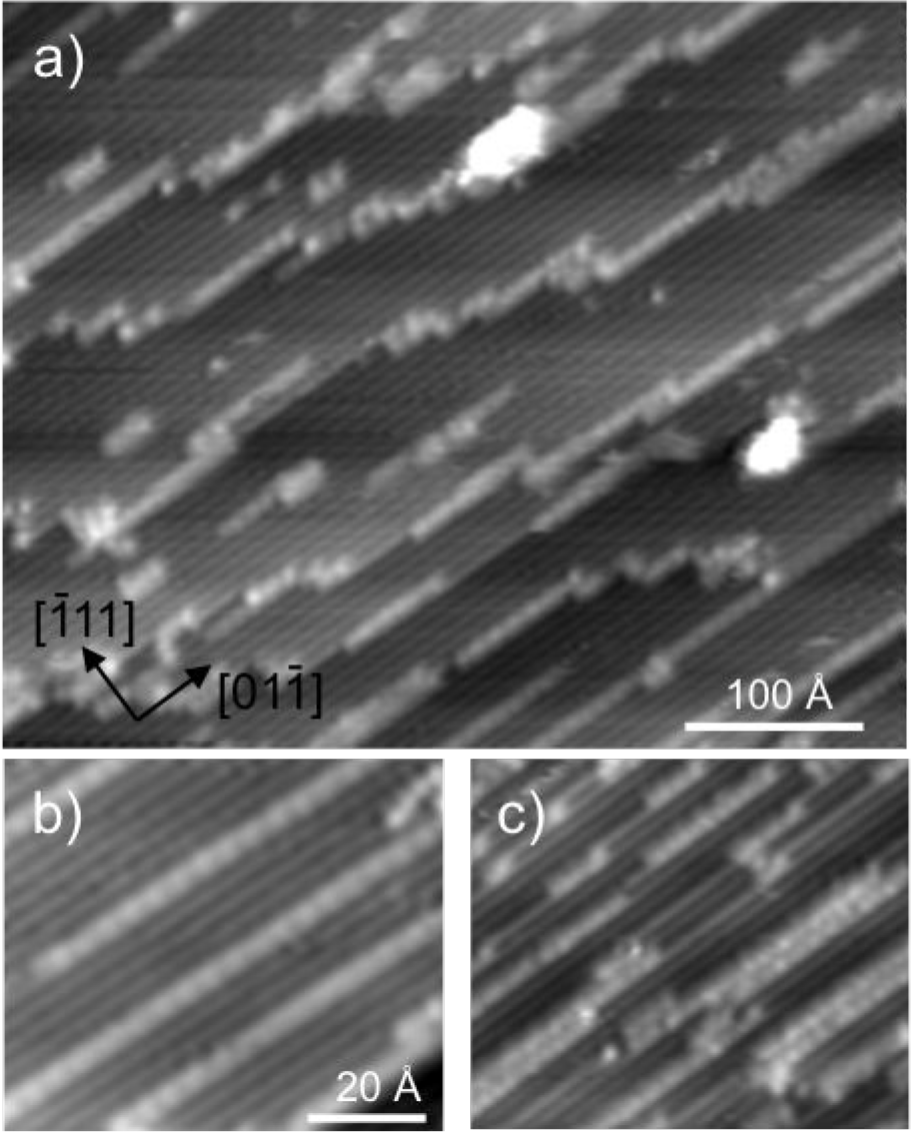
Large scale STM images of a low coverage of water on the Pt(211) surface after annealing to 150 K. The steps run parallel to [011] while [111] is the step-down direction. (a) Image showing water chains running parallel to the steps with water preferentially decorating the top edge of steps between (211) terraces. (b) Water chains formed on a flat (211) terrace with a 4 Pt atom repeat along the step edge. (c) Parallel water rows formed on adjacent steps at a higher water coverage.

Figure 2 shows images of the Pt(211) surface after a small amount of water has been deposited and the surface annealed to 150 K. The surface has large (211) terraces of regular single atom (100) steps, separated by steps that predominantly align along the close-packed [011] direction. Water collects preferentially above steps between (211) terraces, forming narrow, linear structures along the upper step, with similar linear structures also appearing on the (211) terraces, as shown in Figure 2b. Line sections though the water chains are shown in Figure S2, where the propensity to decorate steps between (211) terraces is described in more detail. Although there are some larger clusters formed, the majority of water structures observed are less than one terrace wide (<6.8 Å) and may extend several hundred angstroms along a single Pt step. The water chains formed on Pt(211) terraces are aligned between two parallel steps and show no obvious tendency to aggregate, or cross between adjacent steps, but as the coverage is increased, we start to observe some pairs of parallel water rows formed on adjacent steps, as shown in Figure 2c.

**Figure 3:**
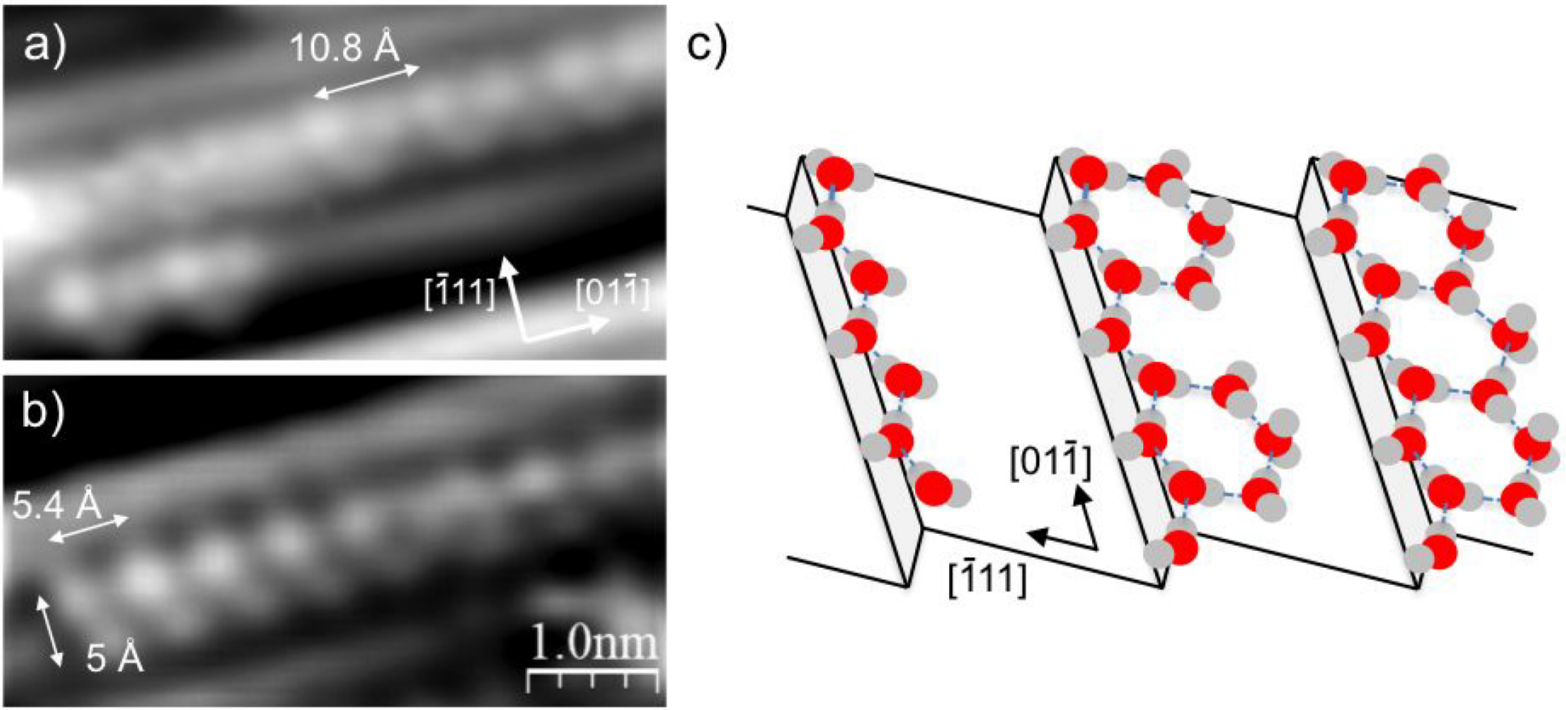
Details showing two types of water chain formed after annealing to 140 K, with (a) showing a repeat every 4 Pt atoms along the step and (b) a 2 Pt repeat. (c) Schematic illustrating structures discussed in the text with (left to right) a zigzag water chain along the step with a 2 Pt repeat, a chain with two hexagonal water rings on the terrace in a 4 Pt repeat, and a complete chain of linked hexagons with a 2 Pt repeat along the step.

Figure 3 shows the structure of the water chains on a (211) terrace in more detail. The chains image as bright features every 5.4 Å (a 2 Pt repeat) along the top of the Pt step, with additional structure on the upper terrace that images slightly fainter, forming triangular features ca. 5 Å wide. The terrace structure is more variable than the features along the step edge, appearing as a bright feature either every 4 Pt sites along the chain (Figure 3a), or else every 2 Pt atoms to form a regular zigzag structure, as shown in Figure 3b. We did not find evidence of water chains without any additional structure on the adjacent terrace, indicating that this water is an integral part of the structures formed and essential to the structure’s stability. The double period of the bright features along the top of the Pt step is consistent with that reported earlier for water on Pt(211) by SXRD and EXAFS and ascribed to linear 2-coordinate water chains^30,31^ in a single donor-single acceptor arrangement, illustrated schematically on the left in Figure 3c. In this model water forms a zigzag water chain atop the Pt step, with alternate water molecules having one uncoordinated H atom that points out over the step, or down toward the terrace, depending on the exact calculation.^27,28,33−36^ This type of chain is consistent with only one of the water molecules appearing bright in STM, giving the double period along the step.

The length of the repeat along the Pt step (5.4 Å) and the width of the structures (5 Å) suggest an H-bonded ring is formed on the zigzag chain, increasing the average water coordination number and hence the overall binding energy. Calculations by Kolb et al.^28^ suggest that decorating a zigzag chain on the step with attached rings of different size on either the upper or lower terrace helps to stabilize water compared to the simple 2 coordinate chain. We do not see any evidence for additional structure on the lower terrace in STM, but the large Pt step corrugation means we cannot exclude the possibility of water decorating the sites immediately below the step, as was observed at 5 K on a Ni(111) step by AFM.^48^ Because we cannot determine the number of water molecules in the rings from these STM images alone,^49^ interpretation of the exact structure of the chains remains tentative. The most obvious possibility to explain the 2 Pt zigzag repeat structure observed along the Pt(211) terraces (Figure 3b) is a face-sharing hexagonal ring, flattened and elongated along the step to match the Pt close-packed repeat, as illustrated in Figure 3c. In this case two waters are bonded flat on the terrace, and the ring is completed by a final water in a double-acceptor configuration, with one H pointing up to create the bright triangular feature seen on the terraces, giving an average H-bond coordination number of 2.5. Calculations for a hexamer ring on a Pt(533) step^28^ indicate this will fit on a Pt(211) terrace, with O extending 3.7 Å from the step, still 3.1 Å from the next Pt step on Pt(211). In contrast, pentamer rings cannot link to form a zigzag 2 Pt repeat along the step, and larger rings would locate O too close to Pt in the next step (e.g., within 1.2 Å for the heptamer on Pt(533)^28^), forcing water out above the adjacent step edge in a manner that is not observed in our STM images.

The water arrangement in the 4 Pt repeat structure (Figure 3a) is less constrained, although the rings look similar to those of the 2 Pt repeat in STM. The limited order present in the terrace structure, which is considerably more difficult to image than an ordered 2D network on a stepped surface,^39^ suggests disorder along the terrace may help relieve lateral strain created by the elongation of the water chains along the step. Indeed, the majority of the longer chains formed at low coverage have the 4 Pt period, suggesting the gaps between the attached rings (shown in Figure 3a and center of Figure 3c) may help relieve strain along the step direction compared to a linked ring structure (Figure 3b and right of Figure 3c) at the expense of a lower H-bond coordination number. Although the additional terrace structure in the 4 Pt repeat (Figure 3a) appears similar to that of the 2 Pt chains, the size of the attached ring is not as constrained as in the short repeat structure, and we cannot rule out a more complex chain structure. For example, images of water at a Ni(111) step also found structures based around a zigzag chain along the step,^48^ but in this case the zigzag chain forms the edge of an alternating face-sharing pentamer–octamer chain on the upper terrace. Water chains on Pt(211) show a similar zigzag 2 Pt alternation along the step, but although this linear face-sharing double pentamer–octamer chain has been found in several water structures,^39,40,48,50^ it is too wide to fit on a single Pt(211) terrace.

**Figure 4:**
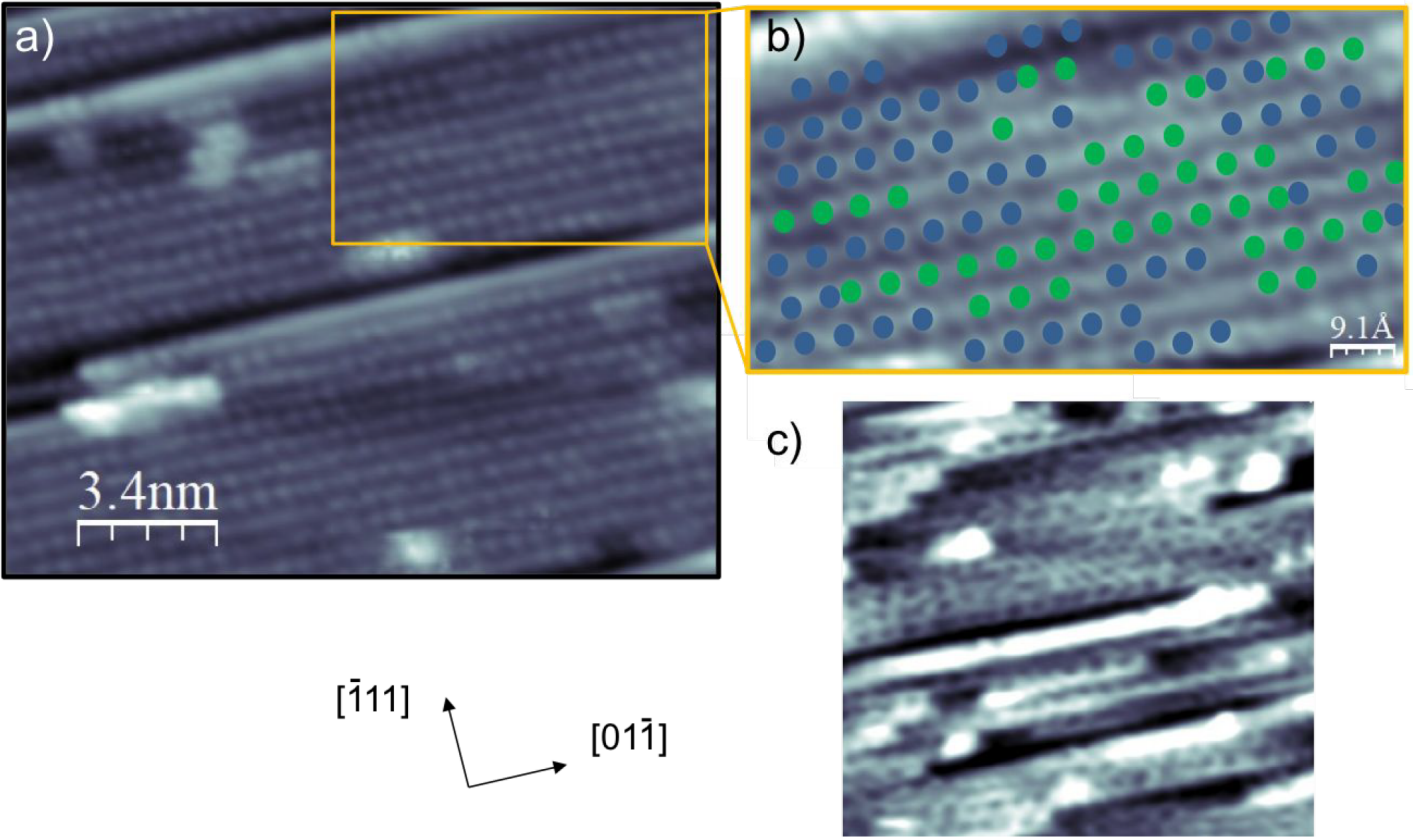
(a) STM image showing the extended structures formed after annealing at 160 K as the water coverage is increased to completely cover the surface. Water forms rows of bright features along the Pt steps with a 2 Pt atom repeat. (b) Section of (a) showing how the phase of the bright features changes between and along the steps, with one phase marked as green dots and the other blue. (c) STM image showing an example of the disordered water network formed as the layer saturates on Pt(211) (ca. 1.1 ML) after excess water is desorbed at 160 K.

As the water coverage is increased, the isolated water rows are replaced by the structure shown in Figure 4a, which covers the entire surface. This structure shows rows of bright features along the Pt steps that mostly retain the double period seen for the low coverage chains, with defects and phase changes along the rows. This is illustrated in Figure 4b, where the phase of the prominent bright features is coded green or blue to highlight the local two times period. Most chains show errors in registry, or phase changes, while the rows of bright features show no obvious ordering from one step to the next, apparently being randomly in or out of phase with each other. This structure appears over a wide range of coverage and is associated with the diffuse half-order structure seen in LEED. The absence of a clear registry between the water on neighboring steps indicates there is no well-defined H-bond linkage between the water rows on adjacent steps, suggesting that this structure consists of the narrow chains similar to those seen at low coverage, packed together along neighboring Pt steps. STM measurements were not able to determine the arrangement of water on the intervening terraces as the images are dominated by the bright features above the step sites. Increasing the water coverage to fully saturate the first layer results in the disappearance of the regular rows as this structure is replaced by a disordered 2D network of water rings, shown in Figure 4c. The water rings in this network sometimes form chains of rings along the step direction, but the ring size is variable, with 2 and 3 atom periods observed locally and other sections show no alignment to the steps. It is no longer possible to identify either the step spacing or any regular period along the steps in STM images of the water network, consistent with the disappearance of the additional LEED beams as the layer completes. It appears that saturation of the surface drives loss of registry between water and Pt, incorporating more water into the first layer, close to the Pt, to form a disordered 2D network of 3-coordinate water in preference to forming second layer water or multilayer water clusters.

Although we are not able to image the terrace structure in the (2 × 1) phase, we can estimate the likely water coverage from the arrangement found in isolated rows at low coverage. An array of water chains along the Pt steps consisting of decorating (4 Pt period) or linked hexagonal rings, with no additional water linking the neighboring rows, would require a coverage of between 0.8 and 0.9 ML water. This picture is consistent with the TDS data that show no change in desorption kinetics until above 0.8 ML coverage (Figure 1) and a water binding energy that is unchanged from the isolated, low coverage chains. If the (2 × 1) phase does contain additional water linking between structures on adjacent steps, this does not increase the stability of the structure. Instead, we find further water adsorption above 0.8 ML drives formation of the incommensurate 2D structure (Figure 4c) causing the binding energy to drop and the TDS peak to shift. Combined with the absence of any clear phase registry between water on adjacent steps, it therefore seems likely the (2 × 1) structure is similar to the isolated rows seen at low coverage and does not have a 2D H-bond network linking the chains together. Although this structure has an H-bond coordination number less than 3, the binding energy achieved by using all of the favored Pt step adsorption sites evidently outweighs the reduced H-bond coordination. The transition to form an incommensurate 2D network as water adsorption saturates the surface (at 1.1 ML, Figure 1) allows more water into close contact with the surface but reduces the water binding energy, shifting the leading edge of the TDS slightly to lower temperature. Loss of the stable water chains along the Pt steps in this structure is presumably offset in part by completion of a 3 coordinate H-bond network, but the system does not support a stable adsorption motif that would lead to a commensurate 2D structure.

The formation of linear water structures and lack of a commensurate 2D structure on Pt(211) are in striking contrast with the formation of ordered 2D phases, not linear chains, on stepped Cu(511), and it is interesting to explore why this should be. Even at low coverage, water on Cu(511) forms ordered 2D islands of interlocking 5-, 6-, and 8-member rings that bridge across the steps.^39^ This network has 3 H-bonds per water, forming short 4-member zigzag water chains along the steps, separated by two vacant step sites; effectively the structure is sacrificing 1/3 of the optimal water binding sites in favor of completing the 2D water H-bond network. The step sites are critical to stabilizing the layer because without the steps the terrace would not wet, but neither are they so strongly binding that the water prefers to fill all these sites at the expense of a reduced H-bond coordination. In contrast, water binds to all of the Pt step sites on Pt(211), forming linear structures that have lower average H-bond coordination number but occupy every Pt step site. The difference between the two systems appears to be driven by the greater binding energy of water on Pt steps compared to Cu, which more than offsets the reduced H-bond coordination on Pt. Different calculations for Cu and Pt stepped surfaces are difficult to compare, but for the (110) surfaces Ren and Meng^51^ find water has a binding energy 0.22 eV/water greater on Pt than Cu, the stronger metal–water bond making it unfavorable to sacrifice occupation of low coordination Pt sites in order to complete the H-bond network.

Increasing the water coverage to fully saturate the Cu(511) surface compresses water into a commensurate 2D hexagonal structure, but this structure again populates only 2/3 of the Cu step sites. On Pt(211) the saturation layer has a water density slightly greater than on Cu and is disordered, making it unclear exactly what fraction of the Pt step sites are filled. The absence of order in the 2D structure formed on Pt(211) is presumably due to the difficulty in forming a commensurate H-bonded structure that both maximizes the water coverage along the steps and matches the Pt step spacing.^52^ In this respect the Cu(511) surface is unusual, having a surface unit cell that matches closely that of bulk ice. Disorder caused by the mismatch between the water–water H-bond length and the spacing of the surface template has also been observed on other plane surfaces, for example, during first and second layer water adsorption on Ru(0001),^50,53^ where the template spacing is shorter than the water H-bond length, and during second layer adsorption on SnPt(111),^40^ where the first layer is rigidly locked to the Pt(111) lattice spacing, causing strain in subsequent water layers.^44^ Whereas these systems relieve strain by forming domain boundaries containing 5- and 8-member water rings, we do not observe any particular motif that relaxes this strain on Pt(211) or allows water to order across the steps. Rather saturation of the layer packs a high density of water onto the Pt surface, maximizing the water–Pt interaction and completing the H-bond network at the expense of occupying all of the Pt step sites.

## Conclusions

Water forms narrow linear structures on Pt(211), forming zigzag chains along the (100) step sites that are decorated on the upper terrace by rings of water. We do not observe simple 2-coordinate water chains, indicating that the increase in the water coordination number above two caused by the additional water on the terrace is essential to the chain’s thermodynamic stability. At higher coverage a disordered structure is formed, containing zigzag water rows along the Pt steps but with no registry between adjacent steps, suggesting it comprises an array of the linear 1D structures. Saturation of the water layer forms a disordered 2D water network containing different ring sizes, maximizing the number of water molecules in contact with the Pt surface and the H-bond coordination at the expense of losing the preferred registry of water along the Pt step sites.

## Supporting Information Available

Additional figures and text showing the surface order determined by LEED as a function of the water coverage and STM line profiles of the water chains (PDF)
