# Supplementary material for: Wetting of a Stepped Platinum (211) Surface
Source: J Phys Chem C Nanomater Interfaces. 2023 Mar 1;127(9):4741–8. doi: 10.1021/acs.jpcc.2c08360 (PMC10009809; doi:10.1021/acs.jpcc.2c08360)
Supplement: Supplementary file 1 — jp2c08360_si_001.pdf [file jp2c08360_si_001.pdf]

Supplementary Information

Wetting of a Stepped Platinum (211) Surface

K. Mistry, N. Gerrard, A. Hodgson*

Surface Science Research Centre and Department of Chemistry, University of Liverpool, Liverpool L69 3BX, UK

** Corresponding Author - Andrew Hodgson, email: ahodgson@liverpool.ac.uk*

Contents

Fig. S1. LEED data showing the surface order as a function of the water coverage.

Fig. S2. Line profiles showing sections through water chains on Pt(211).

Figures

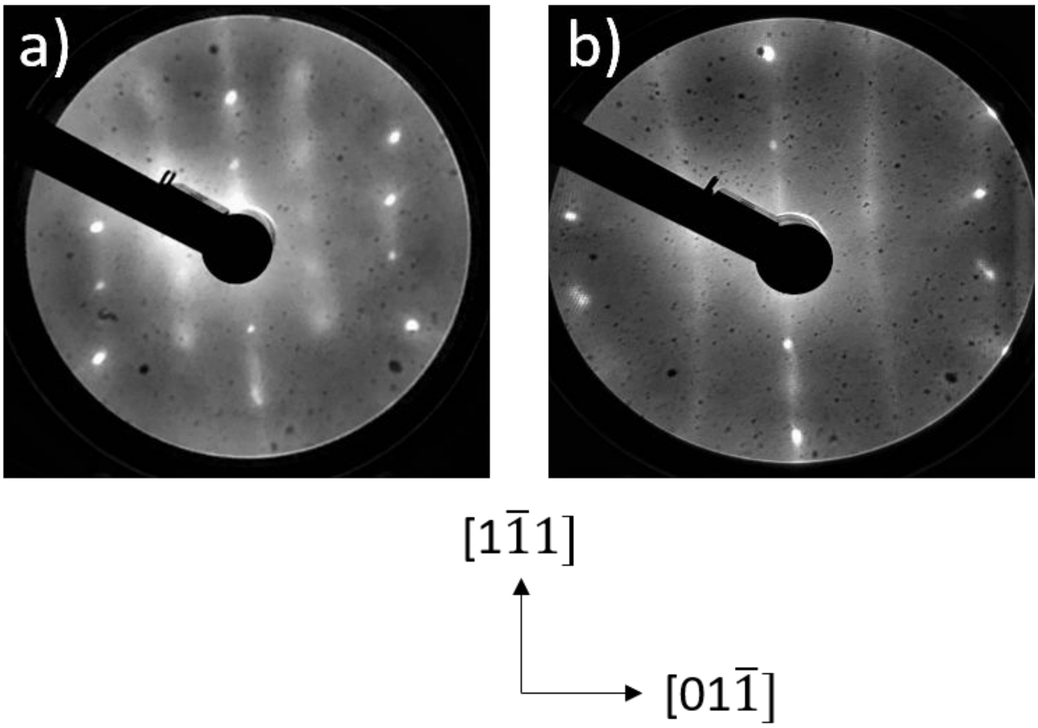

Fig. S1. a) LEED images obtained by annealing ca. 0.4 ML of water to 160 K on the Pt(211) surface, taken at 90 eV, showing diffuse half order spots in the close packed direction. b) LEED image obtained by annealing ca. 0.7 ML of water to 160 K on the Pt(211) surface, taken at 69 eV, showing the half order spots becoming weaker and increasingly streaked in the direction perpendicular to the steps. The images have not been corrected for the radial (pinwheel) distortion caused by the plane channel plate amplifier LEED optics.

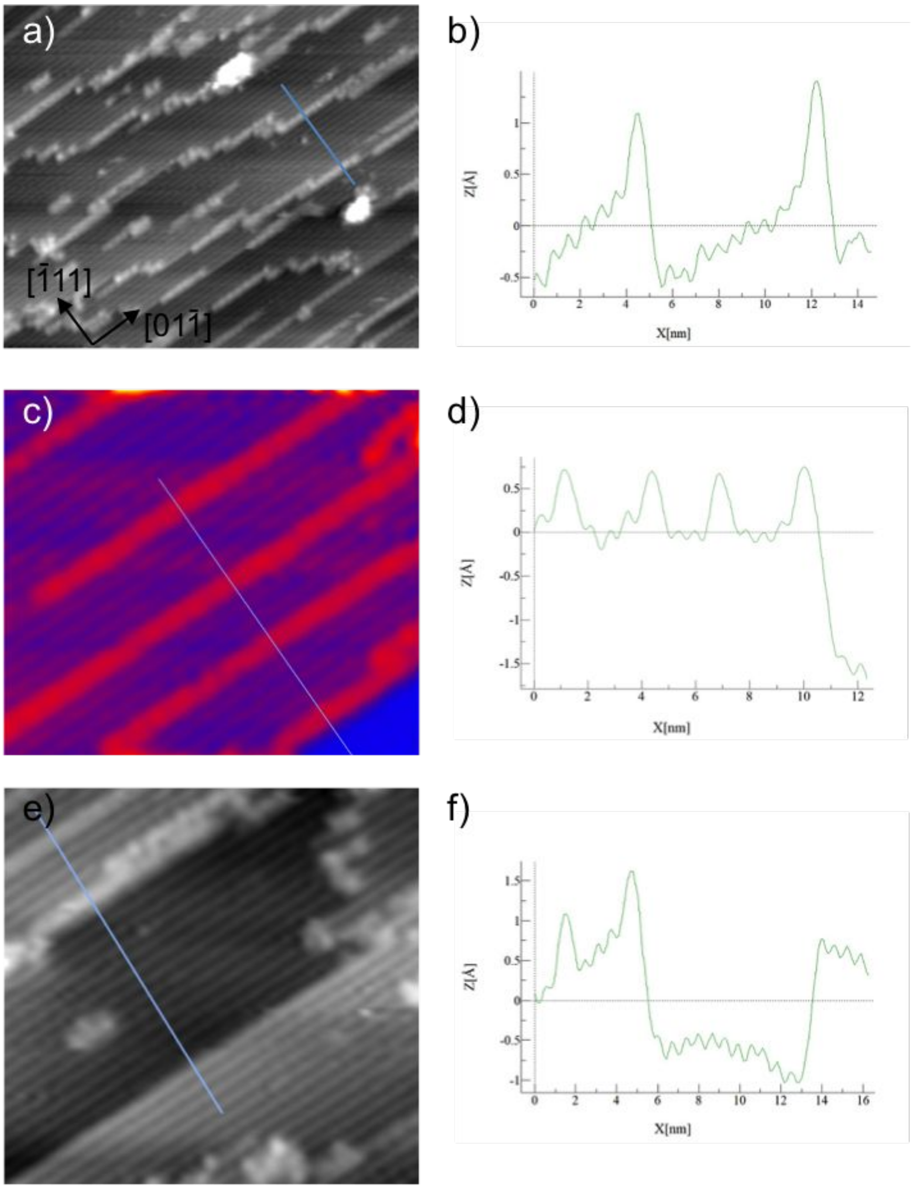

Fig. S2. Line sections showing the profile through the water chains formed on Pt(211). All line sections run from top left to bottom right, along the step-up direction, $[1\bar{1}\bar{1}]$. Frame a) and its line section b) show Pt(211) terraces with the majority of water chains decorating the steps between adjacent (211) terraces. Image c) (Fig, 2c) and its section d) show a series of three chains on the (211) terrace, with the fourth chain decorating the step above a terrace boundary. Frame e) shows a Pt(211) terrace bounded on both sides by a step up to the next (211) terrace. Water preferentially decorates one type of step (top left) but never the other (bottom right), behavior that is repeated across the entire surface. The line section across this terrace f) shows a water chain on the 'flat' (211) terrace to the left, then a water chain decorating the terrace boundary above a down step to the lower Pt(211) terrace, followed by a bare double height step to the right. The (211) terrace boundary that preferentially decorates with water (top left) has a long narrow Pt terrace with a step down on both sides, whereas the step that remains bare (bottom right) has a multiple height step between two (211) terraces.
